# Repair of Tendon Disruption Using a Novel Synthetic Fiber Implant in Dogs and Cats: The Surgical Procedure and Three Case Reports

**DOI:** 10.1155/2020/4146790

**Published:** 2020-07-09

**Authors:** Philippe Buttin, Bastien Goin, Thibaut Cachon, Eric Viguier

**Affiliations:** ^1^Itinerant Surgeon, 471 Chemin de Ronzier, Villaz 74370, France; ^2^Université de Lyon, VetAgro Sup, ICE, Marcy l'Etoile 69280, France; ^3^Novetech Surgery 98000, Monaco

## Abstract

Surgical management of tendon rupture is challenging. One concern is to provide adequate tensile strength to prevent distraction during weight-bearing and gap formation following repair, associated with an increased risk of repair failure. Additional challenges may arise from the nature or the chronicity of the lesion. In the event of avulsion, when the tendon is torn off at the bone insertion, its reinsertion on the bone is generally difficult and may even be impossible in the presence of an avulsion fracture, especially when the bone fragment is too small or fragmented. Repair management is also complicated in chronic cases, as degeneration of the tendon may lead to excessive scar tissue formation, tendon retraction, and muscle atrophy, resulting in a large gap and inadequate tissue for reconstruction. The authors describe the surgical procedure for implanting a novel implant, illustrated by three characteristic clinical cases: (1) an acute Achilles tendon avulsion; (2) a chronic patellar tendon rupture; and (3) a chronic avulsion fracture of the triceps tendon. In these three cases, complete recovery of the function was observed at the last clinical evaluation (6 or 8 months), and no complication was noted. A splinted dressing (6 to 8 weeks) was used successfully in two cases. A resin cast (8 weeks) was preferred in case 1, a very active dog. In conclusion, this novel implant represents a simple procedure for the effective repair of chronic tendon rupture, as well as an effective tendon reinsertion on the bone and adequate support for bone tendon healing in the treatment of tendon avulsion, even in cases of fragmented bone fracture. The thinness of the implant facilitates its insertion into the native tendon, while the bone-screw-implant interface provides immediate and lasting mechanical support. This may facilitate the healing process and potentially shorten the period of immobilization.

## 1. Introduction

Tendon disruption is a common injury in animals that may lead to severe lameness and pain. Rupture can occur at the musculotendinous junction, at the central part of the tendon, or as an avulsion at the enthesis. Surgical repair generally relies on reapposition of the tendon ends or tendon to the bone using various suture patterns.

Surgical management of tendon rupture is challenging. One concern is to provide adequate tensile strength to prevent distraction during weight bearing and gap formation following sutured repair, which will result in reduced long-term strength, thus increasing the risk of repair failure throughout the rehabilitation period [1, 2]. Biomechanically, suture techniques using three-loop pulley and modified three-loop pulley patterns offer higher tensile strength than looking-loop sutures [1, 3, 4]. Nevertheless, sutures do not mechanically resist gap formation [2, 3, 5], especially when subjected to tensile forces associated with locomotion or with rising from a sitting position, when such forces are cyclical [3]. To protect repair, immobilization is, therefore, advocated in order to counteract large tensile force during healing. Several methods including using a cast, splint, screw, and transarticular external skeletal fixator (TESF) are currently used. Lister and colleagues suggested that clinical benefits may result more from decreased weight bearing attributed to the method of immobilization, as well as exercise restriction, as immobilization does not reduce maximum strain on the tendon in a weight-bearing situation [6]. While no difference in clinical outcomes has been found between the use of casts or splints and TESF, complications occur more frequently with the fixation method than with surgical repair [7–9].

A further challenge in surgical management is the nature or the chronicity of the lesion. In case of avulsion, when the tendon is torn off at the bone insertion, its reinsertion to bone is generally difficult and may even become impossible in the presence of an avulsion fracture, especially when the bone fragment is too small or fragmented. Repair management is also complicated in chronic cases, as degeneration of the tendon may lead to excessive scar tissue formation, tendon retraction, and muscle atrophy, resulting in a large gap and inadequate tissue for reconstruction [10]. In human patients, sutured repair of neglected Achilles tendon is augmented using autogenous or exogenous tissues, when the surrounding tissues cannot be used [10–12]. In these cases, augmentation using synthetic grafts has been considered as a satisfactory reconstructive option, associated with an early recovery of the function and a relatively short immobilization period [10]. Recently, a synthetic mesh graft augmentation was used to repair a chronic triceps tendon avulsion in a dog [13]. Reinforcement with synthetic materials offers superior biomechanical properties than suture alone [5, 12], although it does not avoid gap formation following suturing [5]. In order to overcome the problems related to gap formation resulting from chronic degeneration or following sutured repair, while potentially reducing the need for immobilization, Morton and colleagues tested an implant to repair chronic ruptures of the insertion of the gastrocnemius tendon in dogs [14, 15]. The technique involves the fixation of the implant to the calcaneus with an interference screw and provides a better mechanical resistance than suture techniques. According to the authors, such a synthetic implant allows a straightforward surgical procedure that facilitates the reattachment of the tendon, despite excessive scar tissue formation.

In the present paper, the authors describe the surgical procedure for a novel implant for repairing tendon disruption in veterinary patients. The implant is composed of braided grade-medical Ultra-High Molecular Weight Polyethylene (UHMWPE) fibers, a biocompatible material widely used for orthopedic implants in several medical applications [16]. Three characteristic illustrative cases are presented: (1) an acute Achilles tendon avulsion; (2) a chronic patellar tendon rupture; and (3) a chronic avulsion fracture of the triceps tendon.

## 2. Material and Methods

Three patients with acute Achilles tendon avulsion, chronic patellar tendon rupture, and chronic avulsion fracture of the triceps tendon (Table 1) underwent tendinoplasty using the novel implant (Novaten^®^ 8000, Novetech Surgery, Monaco). The decision to perform tendinoplasty was taken in these three cases, as the novel synthetic implant may provide a better option than conventional techniques. Before surgery, the owners provided their written informed consent. They were informed about the proposed surgery, especially about its innovative nature and the lack of clinical follow-up. The risks and expected benefits of this novel procedure, compared to those of conventional approaches, were explained to them.

### 2.1. Perioperative Care

The patients were premedicated with intravenous (IV) morphine (0.2 mg/kg), associated with IV medetomidine hydrochloride (0.015 mg/kg) and ketamine (0.8 mg/kg) for dogs and intramuscular (IM) medetomidine hydrochloride (0.015 mg/kg) and ketamine (4.0 mg/kg) for the cats. Anesthesia was induced with propofol (0.5 mg/kg) and maintained with isoflurane in oxygen. Intravenous cephalexin (20 mg/kg) was also administered preoperatively at 12 hours (25 mg/kg), and then, cephalexin 12.5 mg/kg per os b.i.d. was prescribed for 7 days. Nonsteroidal anti-inflammatory drugs, specifically firocoxib (5 mg/kg), were prescribed during the week following surgery at 1 dose per day and meloxicam for the cat (0.05 mg/kg).

### 2.2. Implant Design and Surgical Technique

**Figure 1:**
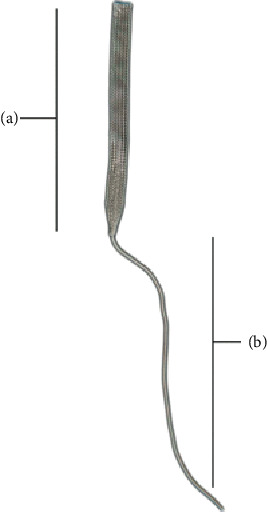
Implant (Novaten®, Novetech Surgery, Monaco). The implant has two components: (a) the implanted section designed to be sutured at its proximal part to the musculotendinous junction and secured at its distal end into the bone using an interference screw; (b) a puller wire allowing the insertion of the implant in bone tunnels.

The implant is composed of UHMWPE fibers, which are biocompatible materials widely used for orthopedic implants in several medical applications, such as anterior cruciate implants [16]. The implant is made of braided fibers, offering superior mechanical and biological properties than free fibers [17], and allows insertion at the exact anatomical insertion site of the native tendon. The implant has two components (Figure 1): the implanted section designed to be sutured at its proximal part to the musculotendinous junction and secured at its distal end into the bone using an interference screw; a puller wire allowing the insertion of the implant in bone tunnels. Two implants and several sizes of drill bits and screws are available to adapt to different patterns of animal osteotendinous structures (Table 2). The implant is placed by sliding into an oblique nonblind tunnel, which facilitates its placement and the adjustment of the tension. When the bone mass is sufficient, the drilling of a second perpendicular tunnel is possible and facilitates the adjustment of the tension by sliding the implant between the two tunnels.

Prior to surgery, the implant was wrapped in a sterile gauze soaked in saline. The affected limb was aseptically prepared, and an adhesive drape was applied. The affected tendons were dissected from enthesis to the musculotendinous junction. When a fibrous scar tissue was observed, it was excised by net incision of the tendon at the limit of this tissue protrusion, to allow adequate healing of the tendon. The proximal end of the tendon was, then, longitudinally incised on half of its diameter, from the musculotendinous junction to the level of the lesion. The implant was placed proximally over the whole length of the half-split tendon, then sandwiched inside the tendon incision, and secured with 8 simple interrupted sutures of 3.5 metric polydioxanone, spaced 5 mm apart, about 4 cm along the implant. An oblique bone tunnel was drilled from the enthesis of the tendon to the plantar or the caudal surface of the bone using a cannulated drill bit on a 2 mm Kirschner wire (corresponding to the screw sizes). In cases 1 and 3, since the size of the bone allowed it, a perpendicular bone tunnel was drilled a few millimeters distally to the exit of the first one, from the lateral to the medial side. In both cases, the entry point was defined in order to preserve cranial and caudal bone margins at least equivalent to the diameter of the screw, to avoid the risk of fracture. The tunnel was tapped. The size of the interference screw was chosen by measuring the depth of the tunnel with a gauge. The implant was inserted in the tunnels via the puller wire by sliding through grommets. Tension and length were adjusted to achieve an appropriate standing angle of the joint. The tension of the implant was, then, maintained using curved Kocher clamps placed at the bone exit. A 1 mm smooth pin was used as a guide to insert the interference screw. The screw was inserted with a ratchet screwdriver, respecting the axis of the pin to avoid the risk of fracturing the trans-cortex. The screw protruded by two threads in order to remain as nontraumatic as possible at the bone-screw-implant interface. The soft tissue and skin were closed routinely.

## 3. Case Reports

### 3.1. Case 1: Achilles Tendon Avulsion

**Figure 2:**
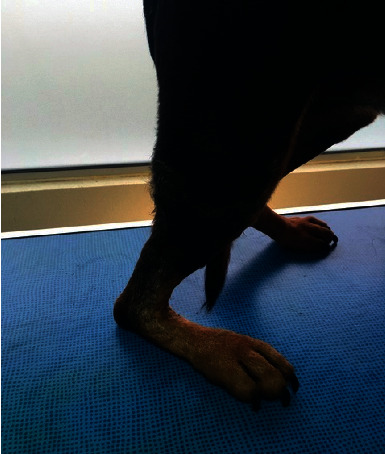
Complete rupture of Achilles tendon in a canine patient with plantigrade stance.

**Figure 3:**
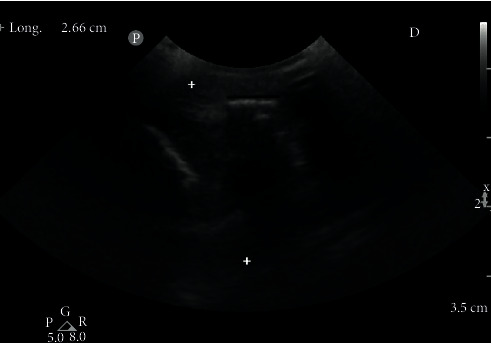
Preoperative ultrasound of a canine patient with acute Achilles tendon avulsion. The black line shows bone tearing at enthesis. Distance between two white crosses shows an abnormally wide thickness of the tendon at the level of rupture.

**Figure 4:**
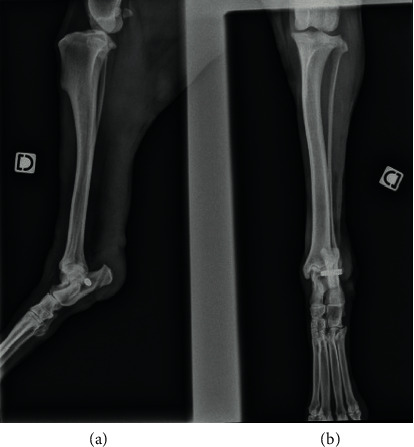
Immediate postoperative lateral (a) and frontal (b) conventional radiography of a canine patient after repair of an acute Achilles tendon avulsion. The interference screw is perfectly positioned. The profile view (a) shows the axis of the bone tunnel outlet at the proximal level of the calcaneus and screw located at the midheight of calcaneus, proximally and caudally to the talocrural joint. The frontal view (b) shows a bicortical characteristic of the interference screw at the level of implantation in calcaneus.

The patient is an eight-year-old Rottweiler (male) weighing 42 kg with a complete rupture of the calcaneal tendon. The initial trauma occurred during hunting one week before referral and caused a tendon avulsion at the calcaneal insertion. The dog had a plantigrade stance (Figure 2) and major lameness. Ultrasound revealed a tendon avulsion with significant desmitis, tendon thickening, and bone tearing at the enthesis (Figure 3). A tendinoplasty using the synthetic implant was decided to facilitate the reinsertion of the avulsed tendon into the bone through the bone-screw-implant interface. There was only mild muscle retraction and no fibrous scar tissue formation at this stage due to the acute nature of the lesion. A caudo-lateral incision was made at the tarsus level and was extended proximally and laterally to the calcaneal tendon up to the level of the musculotendinous junction of the gastrocnemius. The lateral retinaculum was incised and the superficial digital flexor tendon luxated. The proximal ends of the superficial digital flexor and the gastrocnemius tendons were incised on half of their diameter, from the level of the musculotendinous junction of the gastrocnemius to the level of avulsion. The implant was sandwiched inside the incisions of the two tendons and sutured. An oblique 3.6 mm bone tunnel was then drilled in the sagittal plane from the site of avulsion to the plantar surface of the calcaneus with a caudodistal direction. A second 3.6 mm bone tunnel, perpendicular to the first one, was drilled in the calcaneus. The entry point was located at midheight of the calcaneus, proximally and caudally to the talocrural joint. The implant was inserted into the tunnels, and the tension was adjusted to obtain an appropriate standing angle of the tibiotarsal joint, similar to that of the contralateral limb. The required tension was achieved when tarsus resisted flexion during the tibial compression test with the stifle in extension. The implant was then fixed in the transversal tunnel with a 4.5 mm screw. Soft tissue and skin were closed routinely. Immediate postoperative radiographs revealed a perfect positioning of the interference screw (Figure 4). A bivalve cast was applied for 6 weeks. The cast was checked every week and changed every 2 weeks. Over the following 2 weeks, the dorsal valve was removed to allow more movement to the tarsus. The cast was completely removed at 8 weeks.

Upon removal of the cast, the ultrasound revealed normal healing, without effusion of seroma or tendon edema. The dog had a grade II/V lameness. At 9 weeks postoperatively, the owner reported a complete recovery of activity without lameness. The orthopedic examination at 6 months showed no differences between the two hind limbs during gait analysis. No cutaneous fistula was observed. A slight enlargement of the calcaneal tendon enthesis was observed on palpation of the affected limb. Radiographs revealed no bone lysis and a perfect stability of the implant. The owner was completely satisfied with the outcome of the operation.

### 3.2. Case 2: Chronic Patellar Tendon Rupture

The patient is a nine-month-old Maine Coon male (7 kg) which fell from the 4th floor. This initial trauma ruptured the patellar tendon at the middistance between the patella and its tibial tuberosity. The cat first had a tendon suture without external fixation one week after the trauma. The suture finally loosened 2 weeks postoperatively, and the cat had a one-month cage rest. It was, then, received for a second opinion 6 weeks postoperatively. The cat was still limping, and the orthopedic examination revealed weak weight-bearing and a loose patellar tendon. A tendinoplasty was decided in the last intention after the failure of the previous treatment. A cutaneous medial incision was made at the stifle level, followed by a bilateral parapatellar incision of the fascia. An arthrotomy was performed to release the adhesions and assess the position of the patella. Fibrous scar tissue was excised. The half thickness of the tendon was then incised longitudinally from the patella to the musculotendinous junction of the quadriceps. The implant was sandwiched inside the patellar tendon and sutured. A 3.0 mm oblique bone tunnel was drilled through the tibial tuberosity with a caudomediodistal exit. Owing to the small size of the bone, a second perpendicular tunnel was not drilled. The implant was inserted into the tunnel. The stifle was placed in extension, and the tendon was stretched. The implant was temporarily secured with a Kocher clamp to test the resistance during the tibial compression test in extension and 90° flexion. A full range of motion was also tested. The position of the patella was adjusted under direct visualization through the lateral arthrotomy, aiming to achieve correct positioning in the trochlear groove. Needles positioned at the proximal and distal ends of the patella were also used as visual markers. The tension was adjusted before fixation in the tunnel in order to obtain an appropriate standing angle of the stifle and avoid patella alta or baja [18]. A 4.0 mm interference screw was inserted in the oblique tunnel. After fixation, the distal part of the patellar tendon was found to be in excess. This excess of tissue was mainly composed of fibrous tissue and was used to cover the implant at the proximal end of the patella with a Kessler suture. The soft tissue and skin were closed routinely. A splinted dressing was applied for 6 weeks to maintain the stifle in an extended position, with a change of dressing at 2 weeks.

**Figure 5:**
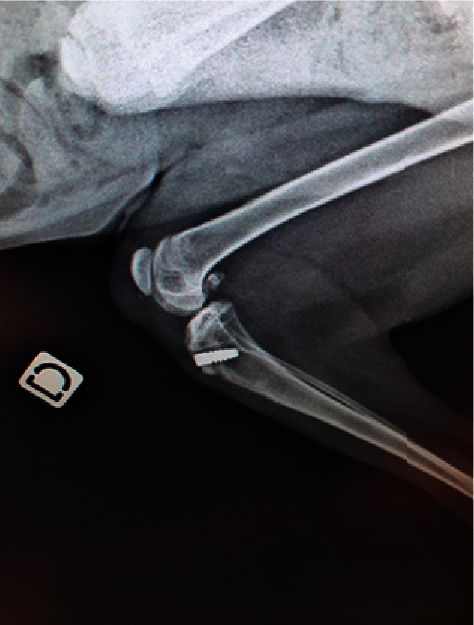
Eight-week postoperative conventional lateral radiography of a feline patient after repair of a patellar tendon rupture, showing the axis of the bone tunnel outlet at the caudal level of tibia and the level of implantation of the interference screw in the tibial crest through metaphysis (almost closed). Note the satisfactory implant position, without enlargement of the bone tunnel at the entry point of the interference screw. Radiolucency at the tip of the screw revealed slight distal displacement, considered as not significant.

When the splinted dressing was removed at 6 weeks, the cat immediately used its hind limb with apparent good weight-bearing and grade II/V lameness. During the 8 weeks following tendon reconstruction, no complication was recorded. The orthopedic examination at 8 weeks revealed a good position of the patella. With the stifle held in extension, the tarsal flexion angle was similar between the two hind limbs. Gait analysis showed a visual symmetric stance phase on both hind limbs and a symmetric carriage at rest in a standing position. Manipulation was pain-free with a full range of motion. Muscle atrophy was still present at this point. The stifle radiography showed a satisfactory implant position, with no patella alta (Figure 5). The last orthopedic examination at 6 months revealed no anatomic or functional difference between the two hind limbs. According to the owner, the cat was able to jump on a table at 2 months postoperatively. The recovery was considered excellent both from the veterinary point of view and the owner's opinion.

### 3.3. Case 3: Chronic Avulsion Fracture of Triceps Tendon

A 7-year-old Cocker Spaniel female (15 kg) with an initial olecranon avulsion fracture was referred two months after the initial trauma, as two successive tension-band fixations had failed. The radiography revealed a fragmentation of the avulsed osseous fragment caudoproximal to the olecranon, which ruled out tension-band fixation. A tendinoplasty was, therefore, decided to reinsert the displaced tendon. An initial surgical procedure was carried out to treat the infection, remove the implants, and debride skin ulcerations. This intervention revealed extensive muscle retraction. After 4 weeks, there was no more infection, and the patient had healed sufficiently to be operated in good conditions. A lateral elbow approach with a skin incision was performed. The anconeus muscle was elevated to reach the olecranon. The dissection was extended proximally to allow individualization of the triceps tendon. Fibrous scar tissue was excised. The half thickness of the tendon was then longitudinally incised up to the musculotendinous junction. The implant was sandwiched inside the triceps tendon and sutured. An oblique bone tunnel was drilled through the olecranon with a caudodistal exit. A second bone tunnel was drilled with a 3.6 mm drill bit in the olecranon, perpendicular and distal to the first one. The entry point was located at the midheight of the ulna, distal and caudal to the humeroulnar joint. The implant was inserted into the tunnels. The elbow was placed in the extension, and the tendon was stretched. The tension was adjusted in order to avoid flexion overstrain before fixation in the perpendicular tunnel. A 4.5 mm interference screw was inserted into the transversal tunnel. The soft tissue and skin were closed routinely. A splinted dressing was applied for 8 weeks to maintain the elbow in an extended position, with a weekly change of dressing.

**Figure 6:**
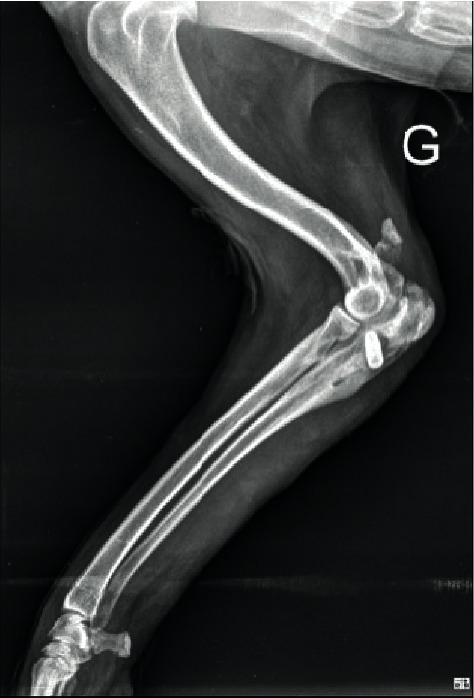
Eight-week postoperative lateral conventional radiography of a canine patient after repair of the chronic avulsion fracture of the triceps tendon, showing the position of the interference screw. The image confirms comminuted avulsion fracture of the olecranon.

**Figure 7:**
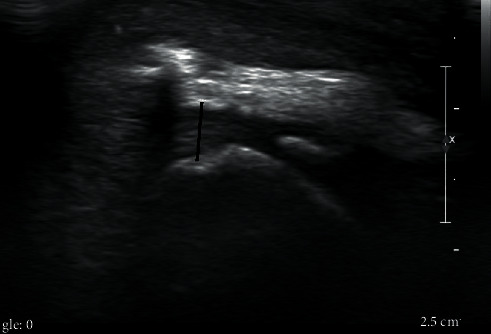
Ten-week postoperative ultrasound of a canine patient after repair of the chronic avulsion fracture of the triceps. The black line shows tendon and implant thickness, revealing good tendon healing and the absence of edema.

During the 8 weeks following tendon reconstruction, no complication was noted. When the splinted dressing was removed at 8 weeks, the dog immediately used his forelimb with apparent good weight-bearing and grade II/V lameness. The orthopedic examination revealed a good tricipital tension at the elbow flexion. Manipulation was pain-free with a full range of motion. Muscle atrophy was still major at this point. There was no change in the implant position on elbow radiography (Figure 6). Ultrasound in week 10 revealed good tendon healing (Figure 7). The last orthopedic examination was performed at 8 months postoperatively, with very good tricipital muscular mass recovery at clinical evaluation. The dog was able to climb mountains with his owner at 4 months postoperatively. Global recovery was considered excellent both from the veterinary point of view and the owner's opinion.

## 4. Discussion

Three cases of tendon disruption were successfully repaired using a novel synthetic implant: (1) an acute avulsion, (2) a chronic patellar tendon rupture, and (3) a chronic avulsion fracture of the triceps tendon. The implant provided effective repair in all cases with relatively early recovery of the function ranging between 2 and 4 months. In all three cases, the decision to perform tendinoplasty was taken because repair with conventional techniques would have been difficult or impossible. In the event of avulsion, when the tendon is torn off at the bone insertion, its reinsertion to bone is generally difficult and may be impossible in the presence of an avulsion fracture, especially when the bone fragment is too small or fragmented. Chronic ruptures are associated with fibrous tissue formation that must be excised to achieve adequate healing. In such cases, a complete excision of fibrous tissue, in addition to chronic muscle contraction, may lead to a large gap formation, making it difficult, if not impossible, to suture the tendon ends. This implant overcomes these problems by bridging the gap and inserting at the exact anatomical insertion site of the native tendon, while providing an immediate and lasting mechanical support with the bone-screw-implant interface.

A similar implant was recently tested by Morton and colleagues in the repair of chronic Achilles tendon ruptures in dogs [14, 15]. However, the technique described above differs from that of Morton and colleagues in three main points. First, this novel implant was slid into a first nonblind tunnel and, then, fixed in a second perpendicular tunnel. The use of a nonblind tunnel facilitates implant placement and the adjustment of the tension. In addition, perpendicular drilling makes it possible to place the screw in a safer area and in the thickness of the bone. Indeed, inserting a screw between two bone plates parallel to the long axis of the bone can cause a fracture if the bone is fragile. According to the authors, inserting the interference screw in a second tunnel perpendicular to the long axis of the bone, ensuring the preservation of cranial and caudal bone margins at least equivalent to the diameter of the screw, limits this risk of fracture. This was considered, particularly indicated in case 3, as the bone was very weakened and fragmented owing to the chronicity of the lesion and several previous surgeries. However, if the size of the bone is not sufficient, drilling a second tunnel may itself carry a risk of fracture that should be considered. This is why the decision was taken in case 2 (a 7 kg cat) to drill only one tunnel in the tibial tuberosity. Secondly, the tendon was incised lengthwise to sandwich the implant. Given the authors' clinical experience, suturing the implant sandwiched inside the tendon is likely to increase tensile strength, thereby offering two possible support points on each side of the implant. A biomechanical study will soon be carried out on this issue. Thirdly, another advantage is that this novel implant is thinner, which facilitates its insertion into the native tendon and sliding into the tunnel, thus saving time and providing more comfort for the surgeon.

Several augmentation techniques using autogenous and allogenous tissues [19–21] or synthetic mesh grafts [5, 10–13] have been described to address the problems associated with chronic tendon ruptures. Among these techniques, the use of synthetic grafts offers the advantages of providing a simple operative procedure with no need to harvest the donor material and reducing the risk of complications such as donor site morbidity, while allowing for early functional recovery and a relatively short immobilization period [10, 11]. Recently, Ambrosius and colleagues reported the repair of a chronic triceps tendon avulsion in a dog using a synthetic mesh graft augmentation [13]. That case is comparable to our third case, although the latter was complicated by a comminuted fracture. The implant allowed for a simple operating procedure in such a challenging case, with functional recovery after an 8-week period of immobilization using a splinted dressing. Although tendon healing is a long process, with experimentally only 56% of the original strength recovery at 6 weeks, slowly reaching 79% at 1 year after repair in tenotomy models [22], current knowledge suggests that postoperative immobilization for 3 weeks followed by a gradual return to activity allows an acceptable return to function while minimizing the risk of rerupture [23]. However, it is difficult to generalize regarding the required period of immobilization. Indeed, based on clinical experience, the duration of immobilization after triceps tendon repair in dogs mostly ranges from 4 to 8 weeks [8]. In addition, chronicity of the avulsion can affect the process of healing of tendon to bone, which may require prolonged immobilization, as in the case reported by Ambrosius and colleagues which required a 12-week immobilization period [13]. In the present technique, the bone-screw-implant interface provides an immediate and lasting mechanical support, ensuring the transmission of tendon strength directly to the bone, which may facilitate the healing process and shorten the immobilization period. Nevertheless, immobilization is necessary to support the proximal implant anchorage, which was found to be weaker than the distal anchorage in the similar implant described by Morton and colleagues [14]. As suggested above, the proximal anchorage of the implant could be strengthened by suturing the implant sandwiched inside the tendon. Nevertheless, further studies are still needed to conclude on the potential postoperative protective benefits of this proximal anchorage.

The choice of the method of immobilization is of concern in tendon repair in many aspects. The main concern is to provide appropriate support during healing, while limiting the risk of complications, mostly attributed to the fixation method [7–9, 15]. Several methods including TESF, casts, splints, or bandages are currently used and require appropriate management to prevent the risk of complications. A rigid immobilization resulting in the complete rest of the tendon may also be counterproductive, as loading of the tendon is required to stimulate repair and appropriate remodeling of collagen fibers [7, 24, 25]. In contrast, the use of a splinted dressing allows a slight range of motion of the joint, thus promoting healing through movement, as well as preventing the risk of ankylosis and muscle contracture. A nonrigid immobilization using a splinted dressing was considered appropriate in cases 2 and 3. In case 1, a cast was preferred because the dog was very active. The immobilization period ranged between 6 and 8 weeks, and no complications were recorded in the three cases.

Synthetic implants are known to be associated with infectious risks [26–28], which may be increased by such a braided implant, as the open pores facilitate cell migration, and by pre-existing superficial subcutaneous infection [28]. The implant should, therefore, not be placed in an infectious area or on an open wound, and careful asepsis should be ensured during surgery. No infection was observed in the three cases. Despite a residual risk of infection, this novel implant may be considered as an effective alternative to conventional repair techniques, especially in chronic cases. In avulsion fractures, the tension-band technique remains the gold standard treatment. However, it may not allow reinsertion of the tendon on the bone, especially when the bone fragment is too small or fragmented. Several techniques for tendon reinsertion have been published. The one involving an interpositional bone plate grafting supplemented with the bone marrow onto a metallic prothesis has been tested successfully in big dog corpses (range: 26–33 kg) [29]. An implant sutured through multiple knot-tying showed excellent clinical results in goats [30, 31]. The implant described in the present report may also be considered as an effective alternative solution, with the advantages of providing a simpler operating procedure and suiting a large range of animal sizes. However, further studies are needed to evaluate the biomechanics of the repair and long-term clinical outcomes in a larger population.

## 5. Conclusions

In conclusion, this novel implant allows effective repair of a chronic tendon rupture, thereby facilitating a surgical procedure that is inherently challenging because of muscle retraction, muscle atrophy, and exuberant mass of the scar tissue leaving a large gap of inadequate tendon. It also allows effective tendon reinsertion on the bone and adequate support for bone tendon healing in the treatment of tendon avulsion, even in cases of fragmented bone fracture. The thinness of the implant facilitates its insertion into the native tendon, while the bone-screw-implant interface provides immediate and lasting mechanical support, thus facilitating the healing process and potentially shortening the period of immobilization.

## Figures and Tables

**Table 1:** Description of cases.

Animal	Weight (kg)	Nature of tendon injury	Method of immobilization	Immobilization period (weeks)	Delay of recovery of functions (months)	
					Subjective^(1)^ (owner's observation)	Objective^(2)^ (clinical evaluation)
Rottweiler (dog)	42	Acute Achilles tendon avulsion	Resin cast	8	2	6

Maine Coon (cat)	7	Chronic patellar tendon rupture	Splinted dressing	6	2	6

Cocker Spaniel (dog)	15	Chronic avulsion fracture of the triceps tendon	Splinted dressing	8	4	8

^(1)^Information collected from the owner. ^(2)^Clinical evaluation during scheduled visit to the veterinarian.

**Table 2:** Novaten® implants and sizes of interference screws and drills.

Implant	Drill size (mm)	Screw diameter (mm)
NOVATEN 4000	3.0	4.0
NOVATEN 8000	3.6	4.5
		5.0

## Data Availability

No additional data are available. All data are included within the article.
